# *Leptospira borgpetersenii* Leucine-Rich Repeat Proteins and Derived Peptides in an Indirect ELISA Development for the Diagnosis of Canine Leptospiral Infections

**DOI:** 10.3390/tropicalmed7100311

**Published:** 2022-10-17

**Authors:** Sineenat Sripattanakul, Teerasak Prapong, Attapon Kamlangdee, Gerd Katzenmeier, Dietmar Haltrich, Ratchanee Hongprayoon, Siriwan Prapong

**Affiliations:** 1 The Interdisciplinary Graduate Program in Genetic Engineering, The Graduate School, Kasetsart University, Bangkok 10900, Thailand; 2 Department of Physiology, Faculty of Veterinary Medicine, Kasetsart University, Bangkok 10900, Thailand; 3 Akkhraratchakumari Veterinary College, Walailak University, Nakhon Si Thammarat 80160, Thailand; 4 One-Health Research Center, Walailak University, Nakhon Si Thammarat 80160, Thailand; 5 Department of Pathology, Faculty of Veterinary Medicine, Kasetsart University, Kamphaengsaen Campus, Nakorn Pathom 73140, Thailand; 6 Department of Food Sciences and Technology, University of Natural Resources and Life Sciences,1180 Vienna, Austria

**Keywords:** leptospirosis, leucine-rich repeat (LRR), ELISA, tropical infectious diseases, emerging and re-emerging infectious diseases, epidemiological surveillance, one health

## Abstract

Domestic and stray dogs can be frequently infected by *Leptospira*, and thus may represent a source for transmission of this zoonotic disease in Thailand. Here, we have used peptides derived from a recombinant leucine-rich repeat (LRR) protein of *Leptospira*, rKU_Sej_LRR_2012M, for the development of an indirect enzyme-linked immunosorbent assay (ELISA) aimed at detecting antibodies against *Leptospira interrogans*, *L. borgpetersenii*, and *L. biflexa*, the three major seroprevalences in Thai dogs. The rKU_Sej_LRR_2012M protein is recognized by hyperimmune sera against several leptospiral serovars. The epitope peptides of the rKU_Sej_LRR_2012M showed binding affinities with lower IC_50_ values than peptides of known antigenic protein LipL32. Four peptides, 2012-3T, 2012-4B, 2012-5B and pool 2012-B, were specifically recognized by rabbit hyperimmune sera against nine serovars from three *Leptospira* spp. The indirect peptide-based ELISAs with these four peptides were evaluated with the LipL32 ELISA by using a receiver–operator curve (ROC) analysis. All peptides had an area under the curve of ROC (AUC) greater than 0.8, and the sum of sensitivity and specificity for each peptide was greater than 1.5. The degree of agreement of 2012-3T and pool 2012-B and 2012-4B and 2012-5B peptides were in moderate-to-good levels with kappa values of 0.41–0.60 and 0.61–0.80, when compared with LipL32, respectively. This finding would suggest an excellent capability of the 2012-4B and 2012-5B peptide-based ELISAs assay for the diagnosis of canine leptospiral infections.

## 1. Introduction

Leptospirosis is a zoonotic disease caused by pathogenic bacteria of the genus *Leptospira* for which recent classifications report 64 species and over 260 serovars [1,2]. In general, signs and symptoms for leptospirosis can range from subclinical to severe clinical signs and death. The general symptoms in dogs are fever, anorexia, conjunctival suffusion, vomiting, myalgia, abdominal pain, jaundice, renal failure, hemorrhage, liver and pulmonary diseases. Signs and symptoms of an infection can range from asymptomatic to severe and even death, depending on the serotype of *Leptospira* and the host [3]. The estimated number of annual cases in humans is 1.03 million, including 58,900 fatal cases worldwide [4]. The highest and lowest number of cases and deaths of leptospirosis in the Thai population from 2006–2020 were around 1638–5439 cases and 24–73 deaths per year [5]. Because of Thai culture, people bigheartedly provide food to stray dogs and the stray dogs live in the public areas in the community. Thus, Thai people stay in close contact to both family and stray dogs, especially in rural areas. Therefore, *Leptospiral*-infected dogs would be prone to carry this zoonotic disease to the Thai population.

The estimated population of dogs in Thailand was around 7 million in 2016, comprising 6.6 million owned dogs and 750,000 stray dogs [6]. The stray dogs have become a public health concern in Thailand, since the numbers increase annually [7]. Stray dogs in Thai urban cities caused noise pollution, road accidents, dog attacks, garbage scavenging, disease transmission (e.g., rabies and leptospirosis), nonhygienic and other problems. The seroprevalence of *Leptospira* of stray dogs in Bangkok was as high as 83.5%, as observed by the microscopic agglutination test (MAT) [7]. Stray dogs are considered an important reservoir of leptospirosis [8,9,10]. Due to their habits, dogs are top predators of many rodents, which are a common reservoir of leptospirosis [11]. In addition, scavenging garbage, smelling urine and licking female genitalia are ways for the transmission to other animals and the environment [10]. Leptospiral infection in dogs can be subclinical or display mild clinical signs, such as fever, lethargy, vomiting and anorexia [11]. Some infections can lead to severe symptoms, e.g., jaundice, kidney, liver and pulmonary failure and death [12].

The prevalence of *Leptospirosis*, analyzed by antisera detection with either microscopic agglutination test (MAT), latex agglutination test (LAT) or ELISA, varies from 4.3 to 89.1% in the Thai dog population [7,13,14,15,16,17,18,19]. However, the prevalence of presenting *Leptospira* pathogens in Thai dogs ranged from 0.37–6.89% and from 0.54–10.3%, when the antigens were investigated by culturing and molecular diagnosis (e.g., PCR), respectively [13,14,20]. Data for the exact incidence of *Leptospirosis* in Thai dog populations show inconsistency depending on the identification method used.

Although the MAT is still considered as a gold standard method, it is very tedious, time consuming and cost-intensive for the cultures and maintenance of *Leptospira* reference strains. The PCR method is a very sensitive technique. It is a useful diagnostic tool when the animals have an acute leptospirosis and a phase of leptospiremia. The latex agglutination test (LAT) and ELISA techniques are very effective and convenient to handle and to identify antibodies against *Leptospira* in serum and plasma samples from animals who had either been infected previously or in acute leptospirosis. However, the suitable antigens for ELISA development must be selected thoroughly, since the commercial vaccines used for veterinary purposes are usually whole cell leptospiral vaccines.

The whole-cell leptospiral vaccines could cause some cross reactivities in vaccinated animals with some major immunogenic outer membrane proteins, such as LipL32, LipL41, LigA, LigB, FcpA and Ompl1 proteins, which have been found in a wide range of immune responses in vaccination experiments and in infected animals [21,22,23,24,25]. Therefore, the development of an ELISA utilizing *Leptospira* proteins, which are expressed mainly after an infection, would offer the prospect of an effective diagnostic tool. Some leptospiral proteins, such as LigB and OmpL37, were reported to show upregulated expression in vivo, where they play a role in the early invasion processes either by binding to host cells EMC proteins or by molecular mimicry process [26,27,28].

The molecular mimicry is a well-known process by which leucine-rich repeat proteins (such as LRR20) of the pathogen compete with the functions of the host to adhere to and invade the host cell [29,30,31]. Proteins containing leucine-rich repeats (LRRs) have been predicted and reported to function in bacterial host–pathogen interactions, membrane anchoring and invasion, such as proteins Internalin A, B, YopM, and LRR20 [30,32,33,34,35,36,37,38,39]. Therefore, *Leptospiral* LRR proteins may be of interest as candidates for the development of *Leptospirosis* diagnostic tools.

Recently, bioinformatics studies revealed that the pathogenic *Leptospira* strains possess more leucine-rich repeat (LRR) genes than non-pathogenic strains [40,41]. There is an LRR protein-encoding gene, LBJ_2012 (Accession # ABJ76523), which was bioinformatically discovered from *L. borgpetersenii* serovar Hardjo-bovis strains JB197 genome and the gene was predicted to encode a hypothetic LRR protein [41]. The LBJ_2012 protein contains the LRR motif of the SD22-like subfamily, which is in internalin A (InA), InB, InC, InH, InC2, InD and InF proteins [41,42].

The LBJ_2012 orthologous LRR coding genes were identified and cloned from multi-cultural passages of *L. borgpetersenii* serovar Ballum, Javanica, Mini, Tarassovi and Sejroe genomes [42]. The *L. borgpetersenii* serovar Sejroe clone R21-2012 truncated leucine-rich repeat protein (LRR) gene, partial cds (Accession # JN627495.1), was found to be naturally inactive, since it contains an internal stop codon [42]. The N- and C-termini of R21-2012 protein, R21-N2012 and R21-C2012, were individually immunoreactive with rabbit hyperimmune serum anti-Sejroe and anti-Javanica, respectively, whereas both peptides immunoreacted with rabbit hyperimmune serum anti-Ballico [42]. However, a membrane lysate, prepared from multi-cultural passages of *L. borgpetersenii* serovar Sejroe, was not immunoreactive with rabbit hyperimmune serum anti the R21-N2012 protein.

The *L. borgpetersenii* serovar Sejroe clone R21-N2012 (Accession: JN627494.1) was mutated and fused with clone R21-C2012 (Accession: JN627492.1) to create the recombinant LRR protein, named rKU_Sej_LRR_2012M [42,43]. In this study, the LRR protein rKU_Sej_LRR_2012M of *Leptospira* was evaluated as an antigen for the development of an indirect ELISA and for the analysis of canine antisera against *Leptospira.* Our objectives were to develop an indirect and a peptide-based ELISA and to analyze canine antisera against *Leptospira*. We have sought to demonstrate antigenic specificity of our recombinant LRR protein and peptides using rabbit hyperimmune sera against nine serovars of *Leptospira* *spp.*

## 2. Materials and Methods

### 2.1. Dog Plasma Samples

Whole blood samples from domestic dogs (*Canis lupus familiaris*) were collected in EDTA tubes (BD Vacutainer K2EDTA; Becton, Dickinson and Company). There were 101 stray dogs and 103 family dogs from the areas of Bangkok (BKK) and Ubon Ratchathani (UBR) province in Thailand, from October to December 2019, with no vaccination history against *Leptospira*. Blood samples were collected from dogs during the sterilization process by the rabies vaccination program organized by the Soi Dog Foundation (Bangkok). All animal protocols for the sample collection were approved by the Kasetsart University Institutional Animal Care and Use Committee (Kasetsart University-IACUC). All client-owned dogs were returned to their owners after spaying and/or rabies vaccination. All stray dogs were released to where they have been trapped by private and public services for spaying/neutering and rabies vaccination. Plasma samples were prepared from EDTA-whole blood samples, according to the standard protocol for plasma preparation and all plasma samples were kept at −40 °C until use. Additional 20 dogs’ sera were received from the sera bank of the Veterinary Teaching Animal Hospital. Dogs were at age of 8–12 months and twice vaccinated by available commercial vaccines against 4 *Leptospira* serovars. All 20 dogs were presented for the neutering program, and all had no history of *Leptospirosis* infection. These 20 sera were assigned as sera from “Vaccinated-dogs”.

### 2.2. Recombinant Proteins

The LipL32 recombinant protein of *L. interrogans* was purchased from Rekom Biotech (Spain). The rKU_Sej_LRR_2012M protein was produced by *Escherichia coli* BL21 Star^TM^ (DE3) expression system, containing the pET161_hKU_R21M_2012 plasmid, as previously described [43,44]. Briefly, the protein expression was induced with 0.1 mM isopropyl β-D-1-thiogalactopyranoside (IPTG; Sigma–Aldrich, Burlington, MA, USA) and incubated at 30 °C, 200 rpm for 12 h. Cells were harvested by centrifugation at 4000× *g* for 15 min at 4 °C and washed with lysis-equilibration-wash buffer (LEW buffer; 50 mM NaH_2_PO_4_, 300 mM NaCl, pH 8.0). The pellet was disrupted using a Misonix XL2020 sonicator (Woburn, MA, USA) with 30% amplitude, a processing time 15 min, 15 s cooling period for each 15 s bursts. The crude lysate was centrifuged at 10,000× *g* for 30 min at 4 °C to collect the inclusion bodies. The insoluble fraction was washed once with LEW and resuspended in denaturing solubilization buffer (DS buffer; 50 mM NaH_2_PO_4_, 300 mM NaCl, 8 M urea, pH 8.0). The solution was centrifuged at 10,000× *g* for 30 min at 20 °C to collect the dissolved proteins. Since the rKU_Sej_LRR_2012M protein was expressed as 6× His-Lumio-TEV fusion proteins, the dissolved proteins were purified by the immobilized metal ion affinity chromatography (IMAC) with Protino^®^ Ni-TED Resin (Macherey-Nagel, Düren, Germany), according to the manufacturer’s protocol with minor modifications, as previously described [45]. The 6× His-Lumio-TEV was cleaved with TEV protease (NEB#P8112, NEB, Ipswich, MA, USA). The protein concentration was determined using a Nanodrop spectrophotometer (Thermo Scientific, Waltham, MA, USA) at 260 and 280 nm. The cleaved and purified protein was analyzed by 12% SDS-PAGE and Coomassie blue staining. The proteins were stored at −20 °C until use.

### 2.3. Epitope Peptide Prediction

The amino acid sequence of the rKU_Sej_LRR_2012M protein was used for the prediction of antigenic epitopes. Linear B-cell epitopes were identified by using online tools, IEDB [46], BcePred [47], BepiPred-2.0 [48], Imed [49], ABCpred [50,51], EMBOSS [52] and SVMTrip [53]. The MHCPred [54,55,56], MHC2Pred [57] and NetMHC [58,59] programs were used for T-cell epitope prediction. Secondary structures of the rKU_Sej_LRR_2012M were analyzed using PHD [60], GOR4 [60], SOPMA [60] and I-TASSER [61,62,63]. Antigenicity scores of B- and T-cell epitopes were evaluated by Vaxijen [64,65,66]. Predicted peptides, obtained from each tool, were aligned with the secondary structures of recombinant proteins. Epitopes with antigenicity scores greater than 0.5 were aligned at coil or loop structures of the protein. Selected epitope peptides were custom-synthesized and purified by the BIOMATIK, Wilmington, DE, USA.

### 2.4. Rabbit Hyperimmune Sera

Rabbit hyperimmune sera against *Leptospira* spp. against 9 serovars of *Leptospira* spp. were purchased from the OIE Reference Laboratory for Leptospirosis and the National Collaborating Centre for Reference and Research on Leptospirosis, the Amsterdam University Medical Centers, Academic Medical Center (AMC), Department of Medical Microbiology and Infection Prevention, University of Amsterdam, (https://leptospira.amsterdamumc.org: access on 5 September 2018). Rabbit hyperimmune sera against the recombinant KU_Sej_LRR_2012M protein (anti-2012) were prepared by the Center for Agricultural Biotechnology using the standard immunization protocol approved by the Kasetsart University Institutional Animal Care and Use Committee (Kasetsart University-IACUC). The non-immunized rabbit serum was used as control serum for ELISA development.

### 2.5. Identification of Leptospira in Plasma Samples by Real-Time PCR

Plasma samples were tested for the presence of *Leptospira* by real-time PCR with Lep F (GGCGGCGCGTCTTAAACATG) and Lep R (TCCCCCCATTGAGCAAGATT) primers [67,68]. Plasma DNA samples were extracted by using phenol: chloroform: isoamyl alcohol (Sigma-Aldrich), as described by Brenner et al. [69] with some modifications as reported by Nitipan [42]. Real-time PCR was performed following manufacturer recommendations. Briefly, 10 µL of a reaction contained 1× iTaq Universal SYBR Green Supermix (Bio-Rad), 300 nmol of each primer and 4.5 µL of an extracted DNA sample. The real-time PCR assay was performed using a CFX96 Touch Deep Well Real-Time PCR Detection System (Bio-Rad) with initial denaturation at 95 °C for 3 min followed by 40 cycles of denaturation at 95 °C for 30 s, annealing/extension at 60 °C for 30 s. The melting curve was analyzed at 60–95 °C, 0.5 °C increments at 5 s/step.

### 2.6. Line Blot Immunodetection of rKU_Sej_LRR_2012M Protein by Rabbit Hyperimmune Sera

Recombinant proteins, rKU_Sej_LRR_2012M and LipL32, at 50 μg/mL were applied to nitrocellulose membranes (Bio-Rad) by a paintbrush. After drying at room temperature, membranes were blocked with 3% BSA in phosphate-buffered saline, pH 7.4 (PBS) for 30 min and washed two times with PBS (pH 7.4) containing 0.05% Tween 20 (PBST). Membranes were incubated with each rabbit hyperimmune sera against *Leptospira* or the rabbit control serum at a dilution of 1:100 for 1 h and 30 min. After washing with PBST, membranes were incubated with goat anti-rabbit IgG (Abcam) conjugated with gold-nanoparticles (0.01 mg of antibody to 1 mL of colloidal gold: Kestrel Bioscience, TH) dilution 1:20 for 30 min. Membranes were washed with distilled water before observing the antigen lines of developed red color gold-nanoparticles on the membrane.

### 2.7. Immunodetection of rKU_Sej_LRR_2012M and Its Derived Peptides by ELISA

The rKU_Sej_LRR_2012M protein and its peptides were immunologically tested with 9 rabbit hyperimmune sera against *Leptospira*, Anti-2012 sera (rabbit hyperimmune sera against the rKU_Sej_LRR_2012M), and the control rabbit serum by an indirect ELISA. An enzyme-linked immunosorbent assay (ELISA) was performed, as described previously with some modifications [70]. The indirect ELISA platforms were optimized by checkerboard titrations. Each peptide (1.25 µg/mL), pool peptides (1.25 µg/mL each) and the rKU_Sej_LRR_2012M protein (7.5 µg/mL) in carbonate-bicarbonate buffer (pH 9.6) was coated on 96-well polystyrene microplates (NuncMaxiSorp, Thermo Fisher Scientific) (100 µL/well) and incubated at room temperature for 2 h. Wells were washed three times with PBST. The wells were blocked with 200 µL blocking buffer (2% BSA in PBST) at room temperature for 1 h in a humid chamber. Next, 100 µL of rabbit serum (each rabbit hyperimmune serum or anti-2012) at a dilution of 1:100 with 2% BSA in PBS or 2% BSA in PBST (as background) was added and incubated at 37 °C for 1 h. After washing, the 100 µL/well chicken anti-rabbit IgG-HRP (Santa Cruz Biotechnology) at a dilution of 1:5000 was added and incubated at 37 °C for 1 h. An amount of 100 µL of the 1-Step^TM^ Ultra TMB substrate solution (Thermo Scientific) was added to each well and incubated for 25 min in the dark. The reaction was stopped with 100 µL/well of 2 M sulfuric acid. Absorbance was measured by an ELISA reader (BioTek^TM,^ Synergy^TM^ H1, Fisher Scientific, Loughborough, UK) at 450 nm. Absorbance of the background was subtracted and samples were compared with the control rabbit serum. All samples were tested in triplicate.

### 2.8. Canine Plasma Samples Leptospira Detection by an Indirect Peptide-Based ELISA

The rKU_Sej_LRR_2012M protein-derived peptides that were ELISA detectable by all rabbit hyperimmune sera against 9 *Leptospira* serovars were chosen for testing with dog plasma samples. The efficacy of the peptide-based indirect ELISA development for each selected peptide was compared to the antigen, LipL32. The 96-well polystyrene microplates (NuncMaxiSorp, Thermo Fisher Scientific) were coated with selected peptides (1.25 µg/mL, 100 µL/well) or LipL32 antigen (1 µg/mL) diluted in carbonate-bicarbonate buffer (pH 9.6) at room temperature for 2 h. The wells of microplate were washed three times with PBST. Wells were blocked with 200 µL of 2% BSA in PBST at room temperature for 1 h. Dog plasma samples at a dilution of 1:100 with 2% BSA in PBS or 2% BSA in PBST (as background) were added and incubated at 37 °C for 1 h. After washing with PBST three times, 100 µL/well goat anti-dog IgG-HRP (Abcam) at a dilution of 1:10,000 was added and incubated at 37 °C for 1 h. An amount of 100 µL/well 1-Step^TM^ Ultra TM substrate solution (Thermo Scientific) were added and incubated for 25 min in the dark. Reactions were stopped with 100 µL/well of 2 M of sulfuric acid. Absorbance of the samples was measured at 450 nm with an ELISA reader (BioTek^TM^ Synergy^TM^ H1, Fisher Scientific, UK). Absorbance of the background was subtracted, and samples were compared with control rabbit serum. All samples were tested in triplicate.

### 2.9. Microscopic Agglutination Test (MAT)

The plasma or sera from 6 positive PCR, 5 positive and 5 negatives evaluated by LipL32, and peptide-based ELISAs or from 20 *Leptospira* vaccinated dogs were validated against *Leptospira* by MAT. The MAT was performed at the University Veterinary College Diagnostic Center by standard method, as explained in Supplementary Data S1.

### 2.10. Statistical Analysis

MedCalc version 20.009 (Ostend, Belgium) was used for statistical analysis of the ELISA and the determination of cutoff values. Cutoff values for ELISA of the epitope peptides were established using a receiver–operator curve (ROC) analysis [71,72]. Each test was evaluated for sensitivity, specificity, positive predictive value (PPV), negative predictive value (NPV) and accuracy (AC), by comparison with ELISA data for LipL32. The degree of agreement between the peptides and LipL32 ELISA assay was determined using kappa value (*K*) with 95% confidence intervals and interpreted as follows: <0.20, poor; 0.21–0.40, fair; 0.41–0.60, moderate; 0.61–0.80, good; and 0.81–1, very good. The non-normally distributed data were evaluated by the Kolmogorov–Smirnov test. Significant differences of each test, compared to positive and negative of LipL32 ELISA results were analyzed using a Mann–Whitney U test. The probability of association between the dogs and the presence of disease were determined using chi-squared test.

## 3. Results

### 3.1. PCR Detection of Leptospira in Dog Plasma Samples

*Leptospira* PCR identification from 204 dog plasma samples showed that samples from 6 dogs were positive, while there were 9 dogs that showed symptoms of leptospirosis. All *Leptospira*-positive dogs were family dogs with clinical signs of leptospirosis. The data are shown in Table 1. Although two stray dogs showed clinical signs, such as leptospirosis, the real-time PCR could not detect the presence of *Leptospira* in their plasma samples.

### 3.2. Line Blots Immunodetection against the Recombinant Protein by Rabbit Hyperimmune Sera

**Figure 1:**
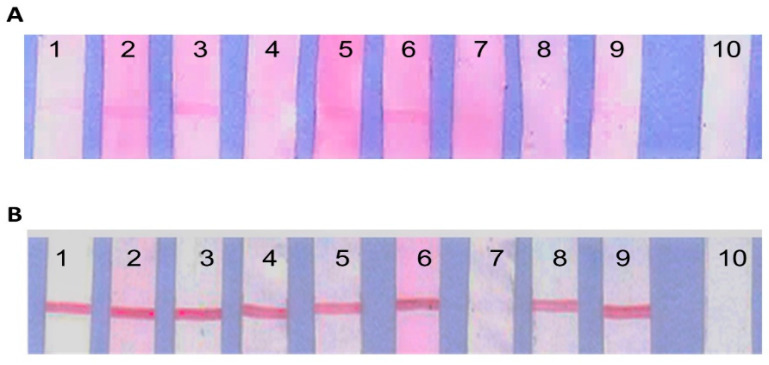
The immunoreactivities of line blot assay between the recombinant proteins, rKU_Sej_LRR_2012M and LipL32. The rKU_Sej_LRR_2012M (**A**) and LipL32 (**B**) at 50 µg/mL with 9 different rabbit hyperimmune sera against *Leptospira* serovar Australis (1), Bataviae (2), Canicola (3), Hebdomadis (4), Icterohaemorrhagiae (5), Mini (6), Patoc (7), Pomona (8), Tarassovi (9) and control or non-immunized rabbit serum (10) at dilution 1:100. Goat anti-rabbit IgG conjugated with gold nanoparticle at dilution 1:20 was used as the immunoreactivity color marker.

The recombinant proteins, rKU_Sej_LRR_2012M and LipL32, were tested with each rabbit hyperimmune sera against nine serovars of *Leptospira* spp., (Table 2), and non-immunized rabbit serum as control serum. The rKU_Sej_LRR_2012M protein immunoreacted with serovars Australis, Bataviae, Canicola, Icterohaemorrhagiae, Mini and Tarassovi, as shown in Figure 1A. The LipL32 was recognized by all serovars of rabbit hyperimmune sera, with the exception of serovar Patoc, as shown in Figure 1B. Both rKU_Sej_LRR_2012M and LipL32 proteins did not immune react with control non-immunized rabbit serum (Figure 1A,B, lane 10).

### 3.3. Prediction on LRR Epitope Peptides

Antigenic epitopes of the rKU_Sej_LRR_2012M protein were predicted by using online tools, and all epitopes were aligned to the corresponding secondary structure of the full-length protein. Four peptides obtained from T-cell epitope prediction and five peptides with predicted B-cell epitopes were selected and commercially synthesized for the peptide-based ELISA development using rabbit hyperimmune sera against nine serovars of *Leptospira* spp. The peptides and their predicted antigenicity scores are shown in Table 3. The selected peptides with high antigenicity scores range from 9–16 amino acid residues, residing from N- to C-terminus of the protein. The highest antigenicity score that was calculated was 1.893 for the peptide 2012-2T and the lowest score was 0.551 for the peptide 2012-4T. The selected predicted B-cell epitopes’ antigenicity scores were highest at 1.672 for the peptide 2012-1B and lowest at 0.836 for the peptide 2012-5B, respectively.

### 3.4. Selection of Antigenic Peptides

**Figure 2:**
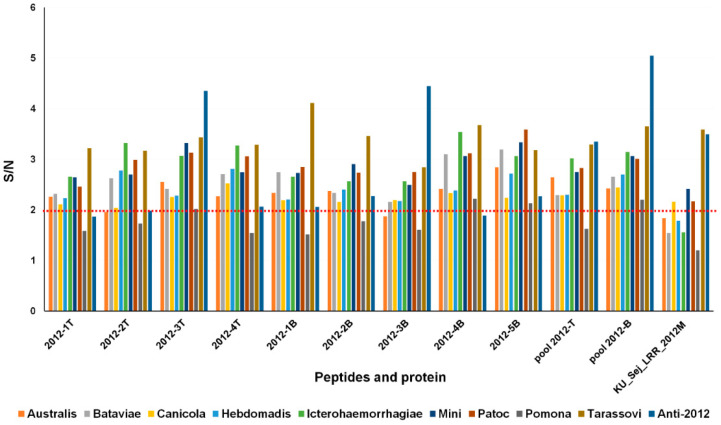
Signal-to-noise (S/N) ratio of the rKU_Sej_LRR_2012M and its derived peptides. The rKU_Sej_LRR_2012M and its peptides were tested with 9 rabbit hyperimmune sera against *Leptospira*, Anti-2012 and the control rabbit serum. The S/N ratio is the ratio of the absorbance of rabbit hyperimmune serum to rabbit control serum (signal per noise; S/N). The cut-off was set at a 2-fold increase in signal over the control. All samples were tested in triplicate.

Four peptides representing T-cell epitopes, five peptides representing B-cell epitopes, pool T-cell epitope peptides (pool 2012-T), pool B-cell epitope peptides (pool 2012-B) and the rKU_Sej_LRR_2012M protein were tested with rabbit hyperimmune sera against nine serovars of *Leptospira* spp., anti-2012, and control rabbit serum. The signal-to-noise (S/N) ratios of each tested peptide or the protein were calculated from the absorbances of each rabbit hyperimmune serum and the control serum, respectively. Numerical data ≥2 than the control for the hyperimmune serum were counted as positive. The recombinant protein rKU_Sej_LRR_2012M was recognized by four serovars of rabbit hyperimmune sera Canicola, Mini, Patoc, and Tarassovi. Peptides 2012-3T, 2012-4B, 2012-5B and pool 2012-B cross reacted with all nine serovars of rabbit hyperimmune sera against *Leptospira*. In addition, the rKU_Sej_LRR_2012M and the peptides 2012-3T, 2012-3B, pool 2012-T and pool 2012-B clearly reacted with anti-2012 (Figure 2). Therefore, the peptides 2012-3T, 2012-4B, 2012-5B and pool 2012-B were selected for peptide-based ELISA detection against *Leptospira* dog plasma samples.

### 3.5. Peptide-Based ELISA of Canine Antisera

**Figure 3:**
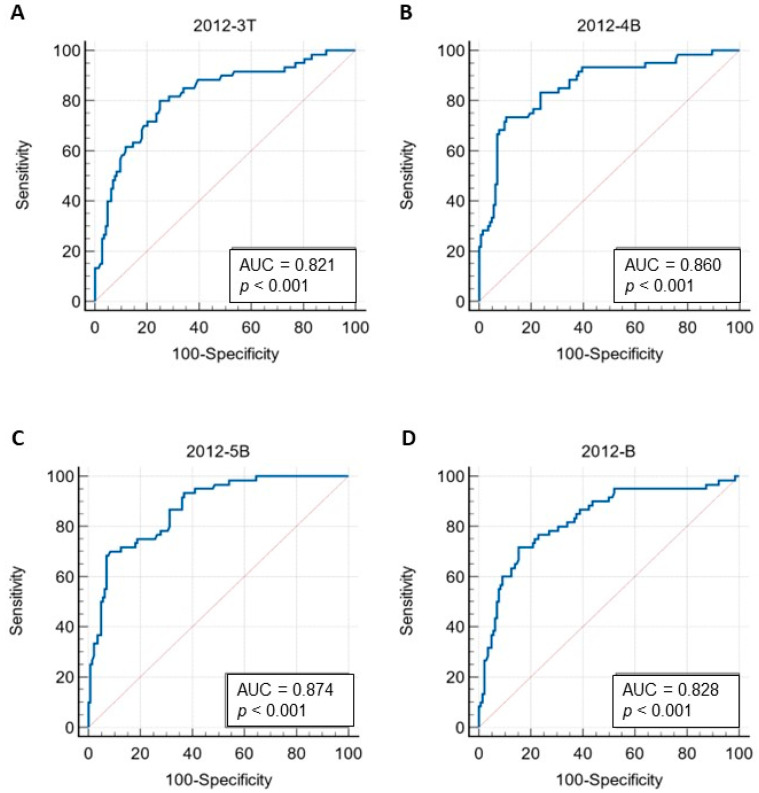
Receiver–operator curve (ROC) and area under the curve of ROC (AUC) of selected peptides tested with dog plasma samples by ELISA, 2012-3T (**A**), 2012-4B (**B**), 2012-5B (**C**) and 2012-B (**D**). ROCs of peptide ELISA were co-analyzed with the ELISA with LipL32 protein. The ROC and area under the curve (AUC) of each peptide were analyzed by MedCalc.

**Figure 4:**
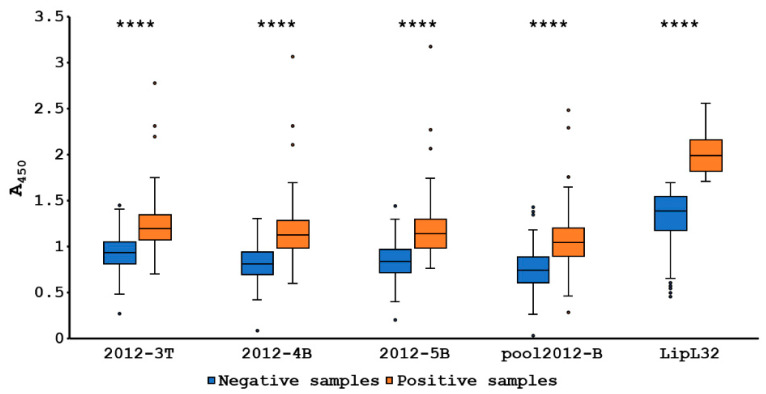
Box plot of absorbance at 450 nm of positive and negative samples of each peptide and LipL32 based on ELISA results. The significant differences between positive and negative results were analyzed using a Mann–Whitney U test (**** represents *p* < 0.0001).

Plasma samples from 204 dogs were immunoassayed against *Leptospira* using peptide-based ELISA with peptides 2012-3T, 2012-4B, 2012-5B and pool 2012-B. The results were compared with antigen protein, LipL32. The cutoff value for each peptide was calculated using a receiver–operator curve (ROC) analysis. The ROC and area under the ROC curve (AUC) of each peptide is shown in Figure 3. The AUC of peptides 2012-3T, 2012-4B, 2012-5B and pool 2012-B was 0.821, 0.860, 0.874 and 0.828, respectively. The cutoff of peptides 2012-3T, 2012-4B, 2012-5B and pool 2012-B was 1.037, 1.020, 1.057 and 0.917, respectively. Six dogs displaying positive results with real-time PCR were also positive with all peptides and LipL32. The performance of each peptide that was co-analyzed with LipL32 ELISA is shown in Table 4. The peptide 2012-3T gave the highest sensitivity followed by peptides 2012-4B, pool 2012-B and 2012-5B, respectively. The peptide 2012-5B exhibited the highest specificity with 0.917. The peptide 2012-5B also presented the highest score for the positive predictive value (PPV). The peptide 2012-5B showed the highest accuracy with 0.853. The negative predictive values (NPV) of peptides 2012-3T, 2012-4B, 2012-5B and pool 2012-B were 0.900, 0.890, 0.880 and 0.878, respectively. The degree of agreement between the peptides and LipL32 were determined by the kappa value. The peptide 2012-3T and pool 2012-B disclosed the moderate agreement with LipL32 with the kappa value at 0.492 and 0.550, respectively. Peptides 2012-4B and 2012-5B were in good agreement with LipL32 with kappa values 0.632 and 0.635, respectively. The Mann–Whitney U test displayed significant differences between the numbers of positive and negative samples of each peptide and LipL32 with *p* < 0.0001 (Figure 4). Peptides 2012-5B and 2012-4B revealed the highest antigenic potential, when compared with other peptides. The prevalence of the *Leptospira* antibody of dogs for 2012-3T, 2012-4B, 2012-5B, pool 2012-B and LipL32 were 41.18% (84/204), 28.92% (59/204), 26.47% (54/204), 31.86% (65/204) and 29.41% (60/204), respectively.

### 3.6. The MAT Validation on the Developed ELISAs Results

Sera from dogs with positive PCR and vaccinated dogs were validated their 2012-4B and 2012-5B peptide-based and LipL32 ELISAs results with MAT results. The 2012-4B and 2012-5B peptide-based and LipL32 ELISAs provided all positive results for all six symptomatic leptospirosis dogs, for which the leptospirosis was confirmed by positive PCR and MAT, as shown in Table 5. All dogs with positive immunoreaction with 2012-4B and 2012-5B peptide-based and LipL32 ELISAs were confirmed by the MAT titer ≥1:100. Although sera from 19 vaccinated dogs were immunopositively detected by the LipL32 ELISA method, only 3sera with MAT titer ≥1:400 to serovar Icterohaemorrhagiae were tested positive by 2012-4B and 2012-5B peptide-based ELISAs (Table 5).

### 3.7. Association between Type of Dogs and Leptospirosis

**Figure 5:**
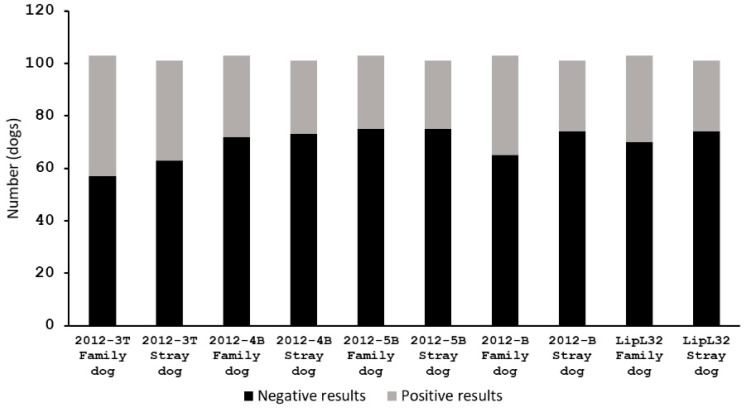
The numbers of dogs that gave positive and negative results from peptide-based ELISA and LipL32 ELISA. The associations between types of dogs (family and stray dogs) and leptospirosis (positive and negative results) were determined using chi-squared test. All data gave *p*-value > 0.05, meaning that leptospirosis was not correlated to the type of dog.

The 103 family dogs and 101 stray dogs in this study were tested for the presence of leptospirosis using the developed peptide-based ELISA and LipL32 ELISA. The number of family dogs that showed positive results with each peptide, 2012-3T, 2012-4B, 2012-5B, pool 2012-B and LipL32, were 46, 31, 28, 38, and 33, respectively, from a total of 103 family dogs (Figure 5). The number of positive results of stray dogs of each peptide, 2012-3T, 2012-4B, 2012-5B, pool 2012-B and LipL32, were 38, 28, 26, 27, and 27, respectively, for a total of 101 stray dogs (Figure 5). The family dogs and stray dogs showed the same incidence of positive and negative *Leptospiral*-associated results. The correlations between types of dogs (family and stray dogs) and leptospirosis (positive and negative results) were determined using chi-squared test. The *p*-values of chi-squared test for ELISA results with peptides 2012-3T, 2012-4B, 2012-5B, pool 2012-B and LipL32 were 0.307, 0.708, 0.815, 0.119 and 0.405, respectively. The *p*-values were greater than the conventional 0.05, the interpretation was that there was no significant relationship between the type of dogs and leptospirosis.

## 4. Discussion

Herein we describe the development of an ELISA using the *Leptospiral* LRR protein KU_Sej_LRR_2012M as an antigen sample. The rKU_Sej_LRR_2012M protein was produced from two overlapping LRR genes of *L. borgpetersenii* serovar Sejroe, the *KU_Sej_R21N_2012* and *KU_Sej_R21C_2012* genes to produce the *KU_Sej_R21_2012M gene* with a deletion at A346 of the gene *KU_Sej_R21_2012* [43]. The gene *KU_Sej_R21_2012* from *L. borgpetersenii* serovar Sejroe genome is an orthologous gene of the *LBJ_2012* gene of *L. borgpetersenii* serovar Hardjo-bovis str. JB197 [42].

Previous studies have shown that the recombinant protein KU_Sej_R21N_2012 demonstrates immunoreactivity against rabbit hyperimmune serum against *L. borgpetersenii* serovar Sejroe and Ballum, and *L. interrogans* serovar Ballico, whereas the KU_Sej_R21C_2012 protein reacted with rabbit hyperimmune serum against *L. borgpetersenii* serovar Javanica, and *L. interrogans* serovar Ballico [42]. Therefore, the rKU_Sej_LRR_2012M and nine corresponding epitope peptides with 9–16 amino acid residues were used for the development of an indirect peptide-based ELISA to detect canine antibodies against *Leptospira*.

Based on the seroprevalence of dog leptospirosis in several regions of Thailand (Supplementary Table S1), the rabbit hyperimmune sera against nine serovars of *Leptospira* spp. were selected for testing by line blots and ELISA. The rabbit hyperimmune sera against serovar Sejroe was not tested with either the line blot or ELISA. Previous observations have shown good immunoreactivity of the KU_Sej_R21N_2012 protein with the rabbit hyperimmune sera against serovar Sejroe [42].

Whereas previous data failed to demonstrate immunoreactivity of the Sejroe rabbit hyperimmune sera with full length rKU_Sej_LRR_2012M protein, our peptide-based ELISA results generated positive results for ELISA detection of serovar Sejroe positive MAT titer from infected dogs.

The line blot and ELISA in this study showed some conflicting observations on the immunoreactivities of the rKU_Sej_LRR_2012M protein with rabbit hyperimmune sera. Authors have observed differences in immunoreactivity between the line blot and the ELISA for the rKU_Sej_LRR_2012M protein, which reacted against serovars Australis, Bataviae, Canicola and Icterohaemorrhagiae, and Mini and Tarassovi in line blot assays and against Canicola, Mini, Tarassovi and Patoc in the ELISA. Since the goat anti-rabbit IgG (Abcam) conjugated with gold-nanoparticles was used as a detector of the line blot assay, it provided higher sensitivity than the ELISA.

The rKU_Sej_LRR_2012M protein immunoreacted against serovar Patoc only in the ELISA method. The different conditions of each method can affect protein folding and, therefore, its binding ability [73]. The rKU_Sej_LRR_2012M protein was dissolved in different buffers and applied onto different supportive materials, which provide different binding strengths and binding abilities. The binding capacity of nitrocellulose membrane is 80–100 µg/cm^2^, while Maxisorp plates have a protein-binding capacity of approximately 600–650 ng IgG/cm^2^. In addition, the rKU_Sej_LRR_2012M protein was used in ELISA at 0.75 μg/reaction, whereas the line blot assay used only 0.125 μg/reaction This means that the number of immunogenic proteins to interact with antibodies on the polystyrene microplate’s well is six times higher than the number of immunogenic proteins on the line membrane.

Although the rKU_Sej_LRR_2012M protein could be detected by rabbit hyperimmune sera against *L. borgpetersenii* serovar Canicola, Mini and Tarassovi in both line-blot and ELISA, the LRR protein could not be detected by rabbit hyperimmune sera against *L. interrogans* serovar Pomona by both techniques. The *LRR gene* sequence of *L. borgpetersenii* serovar Sejroe has 91.4% similarity with the *LBJ_2012* gene of pathogenic *L. borgpetersenii* serovar Hardjo-bovis strain JBL197 (NCBI accession number CP000350 Region: complement [2370828…2371553]). The amino acid sequence of *LBJ_2012* has 97.8% similarity to pathogenic *L. borgpetersenii* serovar Pomona (LEP1GSC133_2670, NCBI accession number EMO64909). Despite considerable similarities within their gene and amino acid sequences, antiserum raised against *L. interrogans* serovar Pomona failed to react with KU_Sej_LRR_2012M protein, thus, indicating a high degree of species-dependent activity. The same serovars of *Leptospira* can be found in multiple species, for example, serovar Pomona not only belongs to *L. interrogans* and *L. borgpetersenii* but also to the species *L. noguchii* [74].

Since the indirect ELISA using the rKU_Sej_LRR_2012M protein could not detect all nine *Leptospiral* serovars, an indirect peptide-based ELISA was developed from the LRR-derived epitope peptides for increasing its efficiency in detecting all of the nine serovars. The peptides of the KU_Sej_LRR_2012M protein; 2012-3T, 2012-4B, 2012-5B and pool 2012-B cross reacted with all nine serovars of rabbit hyperimmune sera against *Leptospira*, though all peptides are predicted antigenic epitopes derived from the rKU_Sej_LRR_2012M protein. These observations are in agreement with the ratios of the number epitopes/well for single peptide or pool peptides to the number epitopes/well for the entire protein, which are 3.3 million and half a million times for the single peptide and pool peptide, respectively. Moreover, working with full-length protein could mask epitopes and generate background signals.

Using these 2012-3T, 2012-4B, 2012-5B and pool 2012-B peptide-based ELISAs to determine immunoreactivity against *Leptospirosis* in dog plasma samples, compared to LipL32 ELISA, the results showed that the AUC of all four peptide-based ELISAs was greater than 0.8, which is considered as excellent [75]. Moreover, the sum of sensitivity and specificity of all peptide-based ELISAs was greater than 1.5, which renders the ELISA a useful test [76]. All peptide-based ELISAs and the LipL32 ELISA gave positive results for the six symptomatic dogs, which they were clarified as leptospirosis by positive PCR and positive MAT tests. Two peptides, 2012-3T and pool 2012-B, showed a moderate agreement (*K* value; 0.41–0.60) with LipL32. Whereas another two peptides, 2012-4B and 2012-5B, showed good agreement (*K* value; 0.61–0.80) with LipL32.

Statistical analysis comparing results from peptide ELISAs to LipL32 ELISA by chi-square showed that the 2012-4B and 2012-5B peptide-based ELISAs provided the lowest false positive. LipL32 is a major outer membrane protein, which was widely studied and developed to be commercial test kits, e.g., SNAP^®^Lepto [77] for the diagnosis of leptospirosis. The LipL32 IgG-ELISA was proved to be sensitive, specific and accurate, as compared to the standard microscopic agglutination test (MAT) in many studies [77,78,79,80]. Therefore, based on the comparison with the LipL32 ELISA, the 2012-4B and 2012-5B are the best peptides to be employed as the peptide-based ELISAs for the analysis of canine antisera against *Leptospira*.

In this study, only six symptomatic dogs (2.94%) were identified positive leptospirosis by real-time PCR assay and MAT, whereas 54 to 84 of 204 dogs (26.47 to 41.18%) including the six symptomatic dogs demonstrated positive results with peptide-based ELISAs. These results are consistent with previous observations having shown that MAT, LAT and ELISA yielded a greater number of positive results than the PCR assay, culture, and microscopic demonstration [13,14,20,81,82]. Unvaccinated dogs may carry *leptospires* in their blood (*leptospiraemia*) for 1–10 days post infection with *L. interrogans* serovar Canicola and for 1–6 days post infection with *L. interrogans* serovar icterohaemorrhagiae [83]. For serological tests, antibodies usually become detectable around the seventh day of the disease [82]. Vaccinated dogs can give positive results with serological test [84]. Healthy dogs tested positive with the LipL32 ELISA, therefore, have previously either been vaccinated or gone on to become infected.

The LipL32 ELISA in this experiment provided positive results in 19 of 20 vaccinated dogs, which is accounts for 95% serological positive in vaccinated dogs. Based on the developed 2012-4B and 2012-5B peptide-based ELISAs, 3 of 20 vaccinated dogs gave positive results, whereas these 3 dogs sera have high MAT titer ≥1:400 for serovar icterohaemorrhagiae. It was counted as 15% positive error by using the 2012-4B and 2012-5B peptide-based ELISAs in vaccinated dogs. The low positive error in the developed peptide-based ELISAs could be results from a naturally additional adenosine residue at position 346 of the *KU_Sej_R21_2012* gene in *L. borgpetersenii* serovar Sejroe genome, when compared to the *LBJ_2012* gene. The addition A346 introduces a stop codon in the middle of the *KU_Sej_R21_2012* gene [42]. The A346 in the *KU_Sej_R21_2012* gene may function to alleviate bacterial pathogenicity during several passages of the culture, which may be a process for vaccine production. Therefore, the screening of leptospirosis in stray dogs by using the developed 2012-4B and 2012-5B peptide-based ELISAs would be applicable for the canine *Leptospira* epidemiological surveillance in Thailand.

Although authors claim that the developed 2012-4B and 2012-5B peptide-based ELISAs are noteworthy, developed kits for the canine *Leptospira* surveillance in Thailand, the rKU_Sej_LRR_2012M protein and its derived peptides should be tested with antisera against other species of *Leptospira*, which have higher seroprevalences in other areas. Since the coverage of 9–10 prevalence serovars in the Thai dog population could not indicate cross immunoreactivities of the protein and its derived peptides with all 260 serovars.

## 5. Conclusions

The KU_Sej_LRR_2012M protein and its derived peptides were tested with rabbit hyperimmune sera against only three species of *Leptospira*. The rKU_Sej_LRR_2012M protein and peptides had potential to detect immunity against *Leptospira* using indirect ELISA, especially 2012-4B and 2012-5B. The 2012-4B and 2012-5B peptide-based indirect ELISAs are noteworthy, developed kits for the canine *Leptospira* surveillance in Thailand. To enhance our understanding of their specificity, the protein and peptides should be tested with antisera against other species of *Leptospira*, therefore, the 2012-4B and 2012-5B peptides and the indirect peptide-based ELISA could possibly be further applied to other domestic animals, such as cattle and pig.

## Figures and Tables

**Table 1:** *Leptospira* identification in dog plasma samples by real-time PCR.

	Symptomatic Dog	Healthy Dog	Positive Real-Time PCR	Total
Stray dog	2	99	0	101
Family dog	7	96	6	103

**Table 2:** The 9 serovars of rabbit hyperimmune sera against *Leptospira* spp. were purchased from the OIE Reference Laboratory for Leptospirosis and the National Collaborating Centre for Reference and Research on Leptospirosis, the Amsterdam University Medical Centers.

Species	Serogroup	Serovar	Strain
*L. interrogans*	Australis	Australis	Ballico
	Bataviae	Bataviae	Swart
	Canicola	Canicola	Hond Utrecht lV
	Hebdomadis	Hebdomadis	Hebdomadis
	Icterohaemorrhagiae	Icterohaemorrhagiae	Ictero l
	Pomona	Pomona	Pomona
*L. biflexa*	Semaranga	Patoc	Patoc l
*L. borgpetersenii*	Mini	Mini	Sari
	Tarassovi	Tarassovi	Perepelitsin

**Table 3:** The predicted antigenic epitopes of the rKU_Sej_LRR_2012M protein-derived peptides.

LRR Protein Derived Peptide Name: Amino Acid Sequence	Length	Sequence Position	Antigenicity Score [Vaxijen]
2012-1T: YKKAGSAAA	9	19–27	0.977
2012-2T: KLSQGTEVR	9	73–81	1.893
2012-3T: EVRFDWLTSL	10	79–88	1.355
2012-4T: KLKELKVPS	9	215–223	0.551
2012-1B: LDEKDSATESN	11	45–55	1.672
2012-2B: KDSATESNIDSLS	13	48–60	1.142
2012-3B: SAPPSDKKLSQGTEVR	16	66–81	0.935
2012-4B: LYPREGKNASK	11	104–114	1.316
2012-5B: GSNNSIKDLS	10	155–164	0.836

**Table 4:** The performance of peptide-based ELISA evaluated with LipL32 antigen results.

Peptide	Cut Off	AUC (95% CI)	Sn	Sp	PPV	NPV	Ac	K (95% CI)
2012-3T	1.037	0.821 (0.754–0.887)	0.800	0.750	0.571	0.900	0.765	0.492 (0.372–0.613)
2012-4B	1.020	0.860 (0.802–0.919)	0.733	0.896	0.746	0.890	0.848	0.632 (0.515–0.750)
2012-5B	1.057	0.874 (0.824–0.924)	0.700	0.917	0.778	0.880	0.853	0.635 (0.517–0.754)
2012-B	0.917	0.828 (0.762–0.893)	0.717	0.847	0.662	0.878	0.809	0.550 (0.426–0.675)

AUC = area under ROC curve; Sn = sensitivity; Sp = specificity; PPV = positive predictive value; NPV = negative predictive value; Ac = accuracy; K = kappa value.

**Table 5:** The validation of peptide-based and LipL32 ELISAs with MAT results from known true positive dogs’ sera.

Methods		Type of Dogs (No. of Dogs with Positive Results)			
		Positive PCR Dogs (6)	Dogs with Positive Results by Peptide-Based and LipL32 ELISAs (5)	Dogs with Negative Results by Peptide-Based and LipL32 ELISAs (5)	Vaccinated Dogs (20)
MAT (serovar with titer ≥ 1:100)	Autumnalis	1	-	-	-
	Bratislava	-	1	-	8
	Canicola	4	2	-	11
	Icterohaemorrhagiae	3	1	-	13 *
	Pomona	-	-	-	6
	Ballum	-	1	-	-
	Sejroe	5	4	-	-
	Tarassovi	4	1	-	-
	Grippotyphosa	-	-	-	3
	Patoc	3	3	-	-
Peptide-based ELISAs	2012-4B	6	5	0	3 *
	2012-5B	6	5	0	3 *
Protein ELISA	LipL32	6	5	0	19

* 3 dogs with MAT titer ≥1:400 to serovar Icterohaemorrhagiae.

## Data Availability

The datasets generated during and/or analyzed during the current study are available from the corresponding author upon request.

## Supplementary Materials

The following supporting information can be downloaded at: https://www.mdpi.com/article/10.3390/tropicalmed7100311/s1, Supplementary Data S1: The microscopic agglutination test (MAT) protocol; Supplementary Table S1: The prevalence of dog leptospirosis in several regions of Thailand.
