# Supplementary material for: Leptospira borgpetersenii Leucine-Rich Repeat Proteins and Derived Peptides in an Indirect ELISA Development for the Diagnosis of Canine Leptospiral Infections
Source: Trop Med Infect Dis. 2022 Oct 17;7(10):311. doi: 10.3390/tropicalmed7100311 (PMC9610812; doi:10.3390/tropicalmed7100311)
Supplement: Supplementary file 1 [file tropicalmed-07-00311-s001.zip › tropicalmed-1930651-supplementary data S1-for proof.pdf]

Supplementary Data S1: The microscopic agglutination test (MAT) protocol

The MAT was performed on all sera by standard method as briefly explain. The living antigens used were 5–7 days old autoagglutination-free cultures grown in Elinghausen McCullough Johnsion Harris (EMJH) medium (Difco, Sparks, MD) with approximately 1×10^8 to 2×10^8 organisms/mL. The MAT was done at doubling dilutions starting from 1 in 25. The reaction was observed after 3 hrs incubation at room temperature by dark field microscopy. Positive samples were titrated up to end titers. A single reciprocal titer of 100 or greater was considered seropositive and indicative of exposure to leptospirosis. A set of 24 reference *Leptospira* serovars was showed in Table below.

The reference serovars and strains of *Leptospira spp.* utilized in MAT.

Genospecies	Serovars[a]	Strain[a]
L. interrogans	Autumnalis	Akiyami
	Bataviae	A Swart
	Bratislava	Jez Bratislava
	Canicola	Houd UtrechtIV
	Djasiman	Djasiman
	Hebdomadis	Hebdomadis
	Icterohaemorrhagiae	RGA
	Pomona	Pomona
	Pyrogenes	Salinem
L. noguchii	Louisiana	LSU 1945
	Panama	CZ 214 K
L. borgpetersenii	Ballum	MUS 127
	Javanica	Veldrat Batavia 46
	Mini	Sari
	Sejroe	M 84
	Tarassovi	Perepelitsin
L. santarosai	Shermani	1342 K
L. weilii	Celledoni	Celledoni
	Manhao	L 105
	Sarmin	Sarmin
L. kirschneri	Cynopteri	3522 C
	Grippotyphosa	Moskva V
L. meyeri	Ranarum	ICF
L. biflexa	Patoc	Patoc 1

[a] The serovars and strains of the *Leptospira* were kindly provided by National Institute of Animal Health, department of Livestock Department, Thailand.
