# Supplementary material for: Leptospira borgpetersenii Leucine-Rich Repeat Proteins and Derived Peptides in an Indirect ELISA Development for the Diagnosis of Canine Leptospiral Infections
Source: Trop Med Infect Dis. 2022 Oct 17;7(10):311. doi: 10.3390/tropicalmed7100311 (PMC9610812; doi:10.3390/tropicalmed7100311)
Supplement: Supplementary file 1 [file tropicalmed-07-00311-s001.zip › tropicalmed-1930651-supplementary Table S1-for proof.pdf]

Table S1. The prevalence of dog leptospirosis in several regions of Thailand.

Region of Thailand	Province	Method of detection	Prevalence	Most common seroreactivity/species	Reference
Northern	Chiang Mai	MAT (≥1:20)	11.0% (23/210)	Bataviae, Canicola, Bratislava, Icterohaemorrhagiae, Ballum, Djasiman, Javanica, Mini, Sejroe	(16)
	Nan	Culture (Urine)<br>*rrs* nested PCR (Urine)	6.89% (4/58)<br>10.3% (6/58)	*L. interrogans*, *L. weilii*	(21)
Northeastern	Chaiyaphum	MAT (≥1:100)	4.26% (2/47)	Autumnalis	(17)
	Mahasarakham	MAT (≥1:100)	10.9% (6/55)	Canicola	(18)
Central	Nakhon Pathom	MAT (≥1:50)	57.5% (88/153)	Tarassovi, Ranarum, Saigon, Bratislava, Copenhageni, Patoc, Bangkok, Sejroe, Autumnalis, Sarmin, Canicola	(19)
	Bangkok	MAT (≥1:100)	89.1% (205/230)	Bataviae, Patoc, Tarassovi, Sejroe, Shermani, Autumnalis, Ranarum, Sarmin, Grippotyphosa, Hebdomadis, Manhao, Pomona, Louisiana, Bratislava, Cynopteri	(8)
Southern	Songkhla	LAT<br>*LipL32* PCR (whole blood)	32.4% (120/370)<br>0.54% (2/370)		(15)
Western	Prachuap Khiri Khan	MAT (≥1:100)<br><br>Microscope (Urine)	28.9% - 42.1% (11-16/38)<br>0% (0/38)	Tarassovi, Bataviae, Sarmin, Canicola, Ranarum, Sejroe, Shermani, Patoc I	(20)
Northern, Northeastern, and Central	-	MAT (≥1:20)	12.1% (33/273)	Anhoa, Australis, Ballum, Bataviae, Bratislava, Broomi, Canicola, Copenhageni, Coxi, Grippotyphosa, Haemolytica, Khorat, Icterohaemorrhagiae, Paidjan, Patoc, Pyrogenes, Rachmati, Saxkoebing, Sejroe	(14)
		ELISA (IgG/IgM)	44.0% (111/252)		
		LipL32 real-time PCR (Urine)	4.39% (12/273)		
		Culture (Urine)	0.37% (1/273)		

Note: MAT = Microscopic agglutination test
LAT: Latex agglutination test
